# Di-*n*-but­yl[4-hy­droxy-*N*′-(3-meth­oxy-2-oxido­benzyl­idene-κ*O* ^2^)benzo­hydrazidato-κ^2^ *N*,*O*]tin(IV)

**DOI:** 10.1107/S1600536813031401

**Published:** 2013-11-23

**Authors:** Yanling Qiao, Fei Wang

**Affiliations:** a College of Chemistry and Chemical Engineering, Liaocheng University, Shandong 252059, People’s Republic of China

## Abstract

In the title compound, [Sn(C_4_H_9_)_2_(C_15_H_12_N_2_O_4_)], the Sn^IV^ atom is coordinated by the N, O and O′ atoms from the tridentate Schiff base dianion in an overall *cis*-C_2_SnNO_2_ trigonal–bipyramidal geometry. Adjacent mol­ecules are linked by O—H⋯O hydrogen bonds, forming a chain running along [001].

## Related literature

For similar organotin compounds, see: Hong *et al.* (2013 ▶).
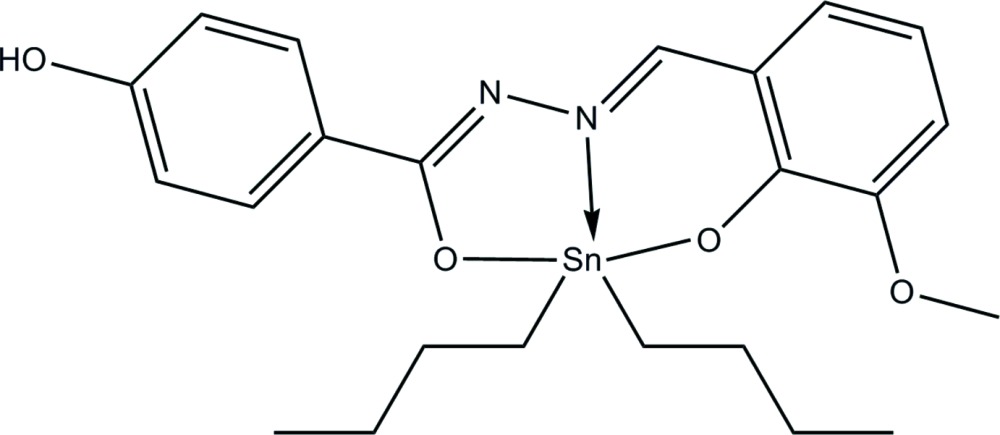


## Experimental

### 

#### Crystal data

[Sn(C_4_H_9_)_2_(C_15_H_12_N_2_O_4_)]
*M* *_r_* = 517.18
Orthorhombic, 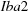

*a* = 10.8504 (3) Å
*b* = 22.2977 (8) Å
*c* = 20.3988 (8) Å
*V* = 4935.2 (3) Å^3^
*Z* = 8
Mo *K*α radiation
μ = 1.06 mm^−1^
*T* = 293 K
0.23 × 0.13 × 0.12 mm

#### Data collection

Bruker SMART 1000 diffractometer
Absorption correction: multi-scan (*SADABS*; Bruker, 2001 ▶) *T*_min_ = 0.792, *T*_max_ = 0.883
16038 measured reflections
4232 independent reflections
3140 reflections with *I* > 2σ(*I*)
*R*_int_ = 0.061

#### Refinement

*R*[*F* ^2^ > 2σ(*F* ^2^)] = 0.039
*wR*(*F* ^2^) = 0.076
*S* = 1.01
4232 reflections
275 parameters
11 restraints
H-atom parameters constrained
Δρ_max_ = 0.60 e Å^−3^
Δρ_min_ = −0.47 e Å^−3^
Absolute structure: Flack (1983 ▶), 1986 Friedel pairs
Absolute structure parameter: −0.03 (3)

### 

Data collection: *SMART* (Bruker, 2007 ▶); cell refinement: *SAINT* (Bruker, 2007 ▶); data reduction: *SAINT*; program(s) used to solve structure: *SHELXS97* (Sheldrick, 2008 ▶); program(s) used to refine structure: *SHELXL97* (Sheldrick, 2008 ▶); molecular graphics: *SHELXTL* (Sheldrick, 2008 ▶); software used to prepare material for publication: *SHELXTL*.

## Supplementary Material

Crystal structure: contains datablock(s) I, global. DOI: 10.1107/S1600536813031401/ng5348sup1.cif

Structure factors: contains datablock(s) I. DOI: 10.1107/S1600536813031401/ng5348Isup2.hkl

Additional supplementary materials:  crystallographic information; 3D view; checkCIF report

## Figures and Tables

**Table 1:** Hydrogen-bond geometry (Å, °)

*D*—H⋯*A*	*D*—H	H⋯*A*	*D*⋯*A*	*D*—H⋯*A*
O1—H1⋯O3^i^	0.82	2.11	2.840 (6)	148
O1—H1⋯O4^i^	0.82	2.26	2.923 (6)	139

Symmetry code: (i) 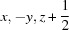.

**Fig. 1.**
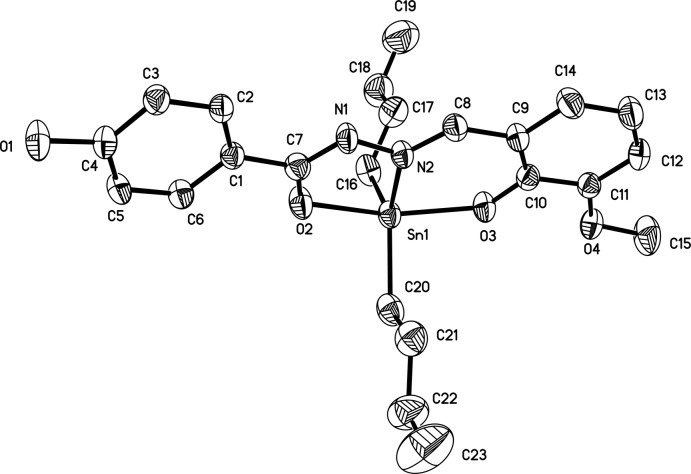
The molecular strucure of compound (I), showing 30% probability displacement ellipsoids. H atoms have been omitted.

**Fig. 2.**
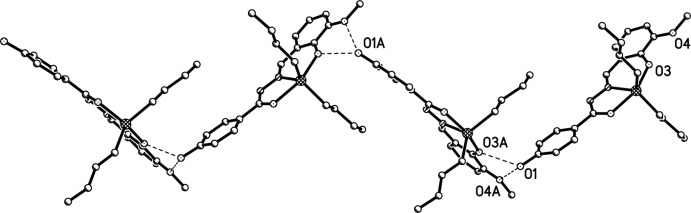
A chain of the compound (I) along c axis, showing the hydrogen bonds as dashed lines. H atoms not involved in hydrogen bonding have been omitted for clarity.

## supplementary crystallographic information

### 1. Comment

The chemistry of organotin(IV) derivatives is a subject of study with growing interest due to their significant antimicrobial properties as well as antitumor activities (Hong *et al.* 2013). As a part of our ongoing investigations in this field we have synthesized the title compound, (I), and present its crystal structure here (Fig. 1).

In the title compound, (I), the Sn atom has distorted trigonal-bipyramidal geometry, with atoms O2 and O3 in axial positions [O2—Sn1—O3 = 155.04 (17) °] and the atoms C16, C20 and N2 in equatorial positions. The sum of the equatorial angles is 359.6 °, indicating approximate coplanarity for these atoms. The Sn1—N2 bond length is 2.176 (5) Å close to the sum of the non-polar covalent radii 2.15 Å, indicating a strong Sn—N interaction. The O atoms coordinate to the Sn atom with one shorter [2.113 (4) Å] and one longer [2.141 (5) Å] bond.

In the crystal structure, intermolecular O—H···O hydrogen bonds (Table 1) link the molecules into a chains along *c* axis (Fig. 2).

### 2. Experimental

[4-Hydroxy-N'-(2-hydroxy-3-methoxybenzylidene)benzohydrazide (1 mmol) and sodium ethoxide (1 mmol) were added to the solution of dry methanol (30 ml) and stirred for 10 mins. Dibutyltin(IV) dichloride (1 mmol) was then added to the reaction and the reaction mixture was stirred for 4 h. The resulting clear solution was evaporated under vacuum. The product was crystallized from a mixture of dichloromethane/ethanol(1:1) to yield orange blocks of the title compound (yield 75%).

### 3. Refinement

The H atoms were fixed geometrically and treated as riding atoms: O—H = 0.82 Å, with *U*_iso_(H) = 1.5 *U*_eq_(O), and C—H = 0.93 - 0.97 Å, with *U*_iso_(H) = 1.2 or 1.5 *U*_eq_(C)

### Crystal data

**Table:**

[Sn(C_4_H_9_)_2_(C_15_H_12_N_2_O_4_)]	*D*_x_ = 1.392 Mg m^−^^3^
*M**_r_* = 517.18	Mo *K*α radiation, λ = 0.71073 Å
Orthorhombic, *I**b**a*2	Cell parameters from 2824 reflections
*a* = 10.8504 (3) Å	θ = 2.7–28.4°
*b* = 22.2977 (8) Å	µ = 1.06 mm^−^^1^
*c* = 20.3988 (8) Å	*T* = 293 K
*V* = 4935.2 (3) Å^3^	Block, orange
*Z* = 8	0.23 × 0.13 × 0.12 mm
*F*(000) = 2112	

### Data collection

**Table:**

Bruker SMART 1000 diffractometer	4232 independent reflections
Radiation source: fine-focus sealed tube	3140 reflections with *I* > 2σ(*I*)
Graphite monochromator	*R*_int_ = 0.061
phi and ω scans	θ_max_ = 25.0°, θ_min_ = 2.7°
Absorption correction: multi-scan (*SADABS*; Bruker, 2001)	*h* = −11→12
*T*_min_ = 0.792, *T*_max_ = 0.883	*k* = −26→26
16038 measured reflections	*l* = −22→24

### Refinement

**Table:**

Refinement on *F*^2^	Secondary atom site location: difference Fourier map
Least-squares matrix: full	Hydrogen site location: inferred from neighbouring sites
*R*[*F*^2^ > 2σ(*F*^2^)] = 0.039	H-atom parameters constrained
*wR*(*F*^2^) = 0.076	*w* = 1/[σ^2^(*F*_o_^2^) + (0.0241*P*)^2^] where *P* = (*F*_o_^2^ + 2*F*_c_^2^)/3
*S* = 1.01	(Δ/σ)_max_ < 0.001
4232 reflections	Δρ_max_ = 0.60 e Å^−^^3^
275 parameters	Δρ_min_ = −0.47 e Å^−^^3^
11 restraints	Absolute structure: Flack (1983), 1986 Friedel pairs
Primary atom site location: structure-invariant direct methods	Absolute structure parameter: −0.03 (3)

### Fractional atomic coordinates and isotropic or equivalent isotropic displacement parameters (Å^2^)

**Table:**

	*x*	*y*	*z*	*U*_iso_*/*U*_eq_	
Sn1	0.81496 (3)	0.065968 (14)	0.62394 (4)	0.05388 (12)	
C1	0.7861 (6)	−0.0474 (3)	0.7979 (4)	0.0495 (17)	
C2	0.6922 (6)	−0.0658 (3)	0.8371 (3)	0.0520 (16)	
H2	0.6119	−0.0545	0.8271	0.062*	
C3	0.7147 (5)	−0.1014 (3)	0.8919 (3)	0.0574 (15)	
H3	0.6494	−0.1147	0.9177	0.069*	
C4	0.8329 (5)	−0.1168 (2)	0.9079 (3)	0.0523 (15)	
C5	0.9281 (5)	−0.0993 (3)	0.8679 (3)	0.0578 (16)	
H5	1.0083	−0.1105	0.8782	0.069*	
C6	0.9064 (5)	−0.0652 (2)	0.8128 (3)	0.0530 (15)	
H6	0.9714	−0.0542	0.7856	0.064*	
C7	0.7658 (6)	−0.0056 (3)	0.7409 (3)	0.0543 (17)	
C8	0.5440 (5)	0.0839 (2)	0.6767 (3)	0.0576 (15)	
H8	0.4820	0.0723	0.7055	0.069*	
C9	0.5110 (4)	0.1286 (2)	0.6287 (4)	0.0529 (13)	
C10	0.5927 (6)	0.1502 (2)	0.5816 (3)	0.0505 (14)	
C11	0.5522 (6)	0.1969 (2)	0.5396 (3)	0.0503 (14)	
C12	0.4334 (6)	0.2188 (2)	0.5439 (3)	0.0631 (17)	
H12	0.4072	0.2490	0.5157	0.076*	
C13	0.3538 (6)	0.1958 (3)	0.5901 (3)	0.077 (2)	
H13	0.2741	0.2109	0.5931	0.092*	
C14	0.3897 (5)	0.1516 (2)	0.6312 (5)	0.0709 (19)	
H14	0.3340	0.1361	0.6615	0.085*	
C15	0.6120 (7)	0.2630 (3)	0.4523 (4)	0.107 (3)	
H15A	0.5454	0.2506	0.4244	0.160*	
H15B	0.6832	0.2718	0.4260	0.160*	
H15C	0.5882	0.2981	0.4763	0.160*	
C16	0.8309 (6)	0.0022 (4)	0.5480 (4)	0.073 (3)	
H16A	0.8822	0.0192	0.5138	0.088*	
H16B	0.8736	−0.0326	0.5652	0.088*	
C17	0.7120 (7)	−0.0187 (4)	0.5172 (4)	0.085 (3)	
H17A	0.6660	0.0156	0.5012	0.102*	
H17B	0.6622	−0.0390	0.5498	0.102*	
C18	0.7394 (9)	−0.0624 (4)	0.4592 (5)	0.108 (4)	
H18A	0.7913	−0.0420	0.4275	0.129*	
H18B	0.7849	−0.0966	0.4757	0.129*	
C19	0.6256 (10)	−0.0842 (5)	0.4254 (6)	0.152 (5)	
H19A	0.5885	−0.1155	0.4509	0.228*	
H19B	0.6469	−0.0994	0.3828	0.228*	
H19C	0.5684	−0.0516	0.4208	0.228*	
C20	0.9550 (5)	0.1273 (3)	0.6486 (3)	0.075 (2)	
H20A	1.0274	0.1048	0.6620	0.090*	
H20B	0.9768	0.1498	0.6096	0.090*	
C21	0.9227 (7)	0.1713 (3)	0.7027 (3)	0.089 (2)	
H21A	0.8995	0.1495	0.7419	0.106*	
H21B	0.8529	0.1956	0.6892	0.106*	
C22	1.0346 (9)	0.2129 (4)	0.7180 (4)	0.133 (3)	
H22A	1.1060	0.1882	0.7276	0.160*	
H22B	1.0534	0.2366	0.6794	0.160*	
C23	1.0110 (13)	0.2544 (4)	0.7752 (5)	0.184 (5)	
H23A	0.9381	0.2776	0.7668	0.276*	
H23B	1.0800	0.2809	0.7806	0.276*	
H23C	0.9998	0.2312	0.8143	0.276*	
N1	0.6566 (4)	0.0188 (2)	0.7374 (2)	0.0579 (14)	
N2	0.6505 (4)	0.05795 (19)	0.6844 (2)	0.0498 (12)	
O1	0.8625 (4)	−0.14853 (19)	0.9631 (2)	0.0720 (12)	
H1	0.8009	−0.1524	0.9860	0.108*	
O2	0.8550 (5)	0.0044 (3)	0.7016 (3)	0.0697 (15)	
O3	0.7075 (3)	0.13012 (17)	0.57351 (19)	0.0609 (11)	
O4	0.6405 (4)	0.21613 (17)	0.4968 (2)	0.0648 (11)	

### Atomic displacement parameters (Å^2^)

**Table:**

	*U*^11^	*U*^22^	*U*^33^	*U*^12^	*U*^13^	*U*^23^
Sn1	0.0519 (2)	0.0615 (2)	0.0483 (2)	0.00729 (17)	0.0015 (4)	0.0122 (3)
C1	0.057 (5)	0.048 (3)	0.043 (4)	0.004 (3)	−0.004 (3)	0.005 (3)
C2	0.048 (4)	0.048 (4)	0.059 (4)	0.001 (3)	−0.003 (3)	0.014 (3)
C3	0.050 (4)	0.061 (4)	0.061 (4)	0.003 (3)	0.005 (3)	0.014 (3)
C4	0.061 (4)	0.050 (3)	0.045 (4)	0.017 (3)	0.003 (3)	0.005 (3)
C5	0.047 (4)	0.072 (4)	0.055 (4)	0.014 (3)	−0.006 (3)	0.007 (3)
C6	0.051 (4)	0.064 (4)	0.044 (3)	0.006 (3)	0.007 (3)	0.007 (3)
C7	0.055 (4)	0.057 (4)	0.052 (4)	0.001 (3)	0.001 (3)	0.001 (3)
C8	0.050 (4)	0.060 (3)	0.064 (4)	0.002 (3)	−0.004 (3)	0.013 (3)
C9	0.049 (3)	0.056 (3)	0.053 (4)	0.010 (2)	−0.005 (5)	0.005 (5)
C10	0.067 (4)	0.043 (3)	0.041 (4)	0.002 (3)	−0.015 (3)	−0.004 (3)
C11	0.060 (4)	0.048 (3)	0.043 (4)	0.003 (3)	−0.010 (3)	−0.004 (3)
C12	0.077 (5)	0.049 (3)	0.064 (5)	0.017 (3)	−0.007 (4)	0.014 (3)
C13	0.061 (4)	0.083 (5)	0.087 (5)	0.018 (4)	−0.002 (4)	0.028 (4)
C14	0.046 (3)	0.084 (4)	0.082 (6)	0.009 (3)	0.003 (5)	0.019 (6)
C15	0.112 (6)	0.111 (6)	0.097 (7)	0.024 (5)	0.002 (5)	0.056 (6)
C16	0.073 (6)	0.081 (6)	0.067 (6)	0.022 (5)	−0.009 (4)	−0.003 (4)
C17	0.106 (7)	0.071 (5)	0.077 (6)	−0.002 (5)	0.001 (5)	0.000 (4)
C18	0.128 (9)	0.100 (7)	0.094 (8)	0.008 (6)	−0.011 (8)	0.008 (6)
C19	0.176 (11)	0.117 (8)	0.162 (12)	−0.043 (8)	−0.049 (10)	0.009 (7)
C20	0.064 (4)	0.098 (4)	0.062 (5)	0.009 (4)	−0.003 (3)	0.017 (4)
C21	0.101 (6)	0.096 (5)	0.069 (5)	0.002 (5)	0.001 (4)	0.013 (4)
C22	0.207 (9)	0.108 (6)	0.086 (6)	−0.052 (7)	−0.029 (6)	−0.003 (5)
C23	0.311 (12)	0.140 (9)	0.101 (8)	−0.055 (9)	0.002 (9)	−0.016 (7)
N1	0.051 (3)	0.062 (3)	0.060 (3)	0.012 (3)	0.000 (2)	0.026 (3)
N2	0.053 (3)	0.053 (3)	0.044 (3)	0.008 (2)	−0.006 (2)	0.008 (2)
O1	0.069 (3)	0.086 (3)	0.061 (3)	0.029 (3)	0.007 (2)	0.030 (3)
O2	0.058 (3)	0.089 (4)	0.062 (4)	0.018 (3)	0.004 (3)	0.029 (3)
O3	0.058 (3)	0.069 (3)	0.055 (3)	0.017 (2)	0.003 (2)	0.015 (2)
O4	0.079 (3)	0.056 (2)	0.059 (3)	0.012 (2)	0.002 (2)	0.014 (2)

### Geometric parameters (Å, º)

**Table:**

Sn1—C20	2.105 (6)	C14—H14	0.9300
Sn1—C16	2.110 (8)	C15—O4	1.418 (7)
Sn1—O3	2.113 (4)	C15—H15A	0.9600
Sn1—O2	2.141 (5)	C15—H15B	0.9600
Sn1—N2	2.176 (5)	C15—H15C	0.9600
C1—C2	1.358 (10)	C16—C17	1.508 (7)
C1—C6	1.398 (9)	C16—H16A	0.9700
C1—C7	1.506 (9)	C16—H16B	0.9700
C2—C3	1.394 (9)	C17—C18	1.561 (12)
C2—H2	0.9300	C17—H17A	0.9700
C3—C4	1.367 (7)	C17—H17B	0.9700
C3—H3	0.9300	C18—C19	1.495 (8)
C4—O1	1.369 (6)	C18—H18A	0.9700
C4—C5	1.373 (7)	C18—H18B	0.9700
C5—C6	1.376 (7)	C19—H19A	0.9600
C5—H5	0.9300	C19—H19B	0.9600
C6—H6	0.9300	C19—H19C	0.9600
C7—O2	1.277 (8)	C20—C21	1.519 (6)
C7—N1	1.307 (8)	C20—H20A	0.9700
C8—N2	1.302 (6)	C20—H20B	0.9700
C8—C9	1.442 (8)	C21—C22	1.558 (10)
C8—H8	0.9300	C21—H21A	0.9700
C9—C10	1.394 (8)	C21—H21B	0.9700
C9—C14	1.414 (6)	C22—C23	1.511 (8)
C10—O3	1.334 (6)	C22—H22A	0.9700
C10—C11	1.419 (7)	C22—H22B	0.9700
C11—O4	1.365 (7)	C23—H23A	0.9600
C11—C12	1.381 (7)	C23—H23B	0.9600
C12—C13	1.379 (8)	C23—H23C	0.9600
C12—H12	0.9300	N1—N2	1.391 (6)
C13—C14	1.351 (9)	O1—H1	0.8200
C13—H13	0.9300		
			
C20—Sn1—C16	123.6 (3)	H15B—C15—H15C	109.5
C20—Sn1—O3	94.31 (19)	C17—C16—Sn1	116.4 (6)
C16—Sn1—O3	98.3 (3)	C17—C16—H16A	108.2
C20—Sn1—O2	95.4 (2)	Sn1—C16—H16A	108.2
C16—Sn1—O2	95.4 (2)	C17—C16—H16B	108.2
O3—Sn1—O2	155.04 (17)	Sn1—C16—H16B	108.2
C20—Sn1—N2	120.7 (2)	H16A—C16—H16B	107.4
C16—Sn1—N2	115.3 (2)	C16—C17—C18	110.2 (7)
O3—Sn1—N2	83.05 (15)	C16—C17—H17A	109.6
O2—Sn1—N2	72.22 (18)	C18—C17—H17A	109.6
C2—C1—C6	119.2 (6)	C16—C17—H17B	109.6
C2—C1—C7	122.1 (6)	C18—C17—H17B	109.6
C6—C1—C7	118.7 (6)	H17A—C17—H17B	108.1
C1—C2—C3	120.8 (6)	C19—C18—C17	113.3 (9)
C1—C2—H2	119.6	C19—C18—H18A	108.9
C3—C2—H2	119.6	C17—C18—H18A	108.9
C4—C3—C2	119.9 (6)	C19—C18—H18B	108.9
C4—C3—H3	120.1	C17—C18—H18B	108.9
C2—C3—H3	120.1	H18A—C18—H18B	107.7
C3—C4—O1	123.1 (5)	C18—C19—H19A	109.5
C3—C4—C5	119.5 (5)	C18—C19—H19B	109.5
O1—C4—C5	117.4 (5)	H19A—C19—H19B	109.5
C4—C5—C6	120.9 (5)	C18—C19—H19C	109.5
C4—C5—H5	119.5	H19A—C19—H19C	109.5
C6—C5—H5	119.5	H19B—C19—H19C	109.5
C5—C6—C1	119.6 (6)	C21—C20—Sn1	115.3 (4)
C5—C6—H6	120.2	C21—C20—H20A	108.5
C1—C6—H6	120.2	Sn1—C20—H20A	108.5
O2—C7—N1	125.4 (6)	C21—C20—H20B	108.5
O2—C7—C1	118.9 (6)	Sn1—C20—H20B	108.5
N1—C7—C1	115.7 (6)	H20A—C20—H20B	107.5
N2—C8—C9	127.5 (5)	C20—C21—C22	110.5 (6)
N2—C8—H8	116.2	C20—C21—H21A	109.5
C9—C8—H8	116.2	C22—C21—H21A	109.5
C10—C9—C14	119.5 (7)	C20—C21—H21B	109.5
C10—C9—C8	123.3 (5)	C22—C21—H21B	109.5
C14—C9—C8	117.2 (7)	H21A—C21—H21B	108.1
O3—C10—C9	124.3 (5)	C23—C22—C21	112.7 (8)
O3—C10—C11	117.5 (5)	C23—C22—H22A	109.0
C9—C10—C11	118.2 (6)	C21—C22—H22A	109.0
O4—C11—C12	125.8 (5)	C23—C22—H22B	109.0
O4—C11—C10	113.5 (5)	C21—C22—H22B	109.0
C12—C11—C10	120.7 (6)	H22A—C22—H22B	107.8
C13—C12—C11	119.7 (6)	C22—C23—H23A	109.5
C13—C12—H12	120.1	C22—C23—H23B	109.5
C11—C12—H12	120.1	H23A—C23—H23B	109.5
C14—C13—C12	121.1 (6)	C22—C23—H23C	109.5
C14—C13—H13	119.4	H23A—C23—H23C	109.5
C12—C13—H13	119.4	H23B—C23—H23C	109.5
C13—C14—C9	120.7 (8)	C7—N1—N2	110.3 (5)
C13—C14—H14	119.7	C8—N2—N1	114.5 (5)
C9—C14—H14	119.7	C8—N2—Sn1	128.5 (4)
O4—C15—H15A	109.5	N1—N2—Sn1	117.0 (3)
O4—C15—H15B	109.5	C4—O1—H1	109.5
H15A—C15—H15B	109.5	C7—O2—Sn1	115.0 (5)
O4—C15—H15C	109.5	C10—O3—Sn1	133.0 (3)
H15A—C15—H15C	109.5	C11—O4—C15	119.2 (5)

### Hydrogen-bond geometry (Å, º)

**Table:**

*D*—H···*A*	*D*—H	H···*A*	*D*···*A*	*D*—H···*A*
O1—H1···O3^i^	0.82	2.11	2.840 (6)	148
O1—H1···O4^i^	0.82	2.26	2.923 (6)	139

Symmetry code: (i) *x*, −*y*, *z*+1/2.
